# Nursing Interventions to Reduce Health Risks from Climate Change Impact in Urban Areas: A Scoping Review

**DOI:** 10.3390/ijerph22081177

**Published:** 2025-07-25

**Authors:** Maria João Salvador Costa, Ulisses Azeiteiro, Robert Ryan, Cândida Ferrito, Pedro Melo

**Affiliations:** 1 Centre for Interdisciplinary Research in Health (CIIS), Faculty of Health Sciences and Nursing, Universidade Católica Portuguesa, 1649-023 Lisboa, Portugal; candida.ferrito@ucp.pt; 2 Centre for Environmental and Marine Studies (CESAM) & Department of Biology, University of Aveiro, Campus Universitário de Santiago, 3810-193 Aveiro, Portugal; ulisses@ua.pt; 3 Department of Landscape Architecture and Regional Planning, University of Massachusetts, Amherst, MA 01003, USA; rlryan@larp.umass.edu; 4 RISE-Health Nursing School of Porto, Universidade do Porto, 4200-072 Porto, Portugal; pedromelo@esenf.pt

**Keywords:** community health nursing, empowerment, patient participation, health promotion, environmental health, climate change, preventive health services, primary prevention

## Abstract

In recent studies, public health has been considered a key stakeholder in climate mitigation and adaptation in cities since they are more exposed to the impact of climate change. Nurses represent a vast majority of public health professionals, playing a key role in health promotion that allows them to influence individuals, families, and communities in adopting healthier behaviours and decarbonized lifestyles. Therefore, the purpose of this study is to map the existing evidence on nursing interventions, which are being led or implemented to reduce the health risks related to climate change in urban areas. The present review follows the JBI methodological framework, including a search on PubMed, MEDLINE complete, CINAHL Complete, Scopus, Web of Science, SciELO (Scientific Electronic Library Online), BASE (Bielefeld Academic Search Engine), and RCAAP. Hand searched references were also considered, including quantitative, qualitative, and mixed-methods studies between January 2014 and October 2024, for a more contemporary perspective. A three-step search strategy and data extraction tool were used by two independent reviewers. Twenty-seven studies in English and Portuguese were eligible for inclusion, all targeting a population of professionals with nursing-related roles: two case studies, one Delphi panel, one descriptive study, one historical research paper, two using a methodological design format, four narrative reviews, one observational study, nine review articles, three scoping reviews, and three systematic reviews. Eight categories of nursing interventions that contribute to decarbonized lifestyles, reducing health risks in relation to climate change, were acknowledged. Nurses play a key role in empowering individuals, families, and communities, promoting climate awareness and literacy, supporting health policy change, advocating for the most vulnerable and engaging in environmental activism, using evidence-based research, and taking advantage of marketing strategies and social media.

## 1. Introduction

Addressing climate change is no longer considered the realm of ecologists and environmental scientists, as “health providers…educators, and leaders, nurses” too are required to ensure communities are well prepared to face the impact of the variability of the climate on human health [1].

Therefore, governments and civil society need to be prepared to face present and future challenges. The Lancet and the University College London Institute (UCL) stated in their earlier report that climate change would not only constitute the most significant global threat of the 21st century but claimed that interventions from all sectors of society would be required to tackle its impact on human health [2].

International discussion regarding climate change began in 1972 in Sweden when the United Nations (UN) First Earth Summit was held and the issue of climate change raised for the very first time [3]. Several Conferences of the Parties (COPs) have been held since, focusing on limiting global warming by 1.5 degrees and pursuing the Paris Agreement goals [4]. More recently, in 2023 in Dubai, participating countries agreed to ensure climate justice by providing new fundings for loss and damage to support the most climate-vulnerable countries. In 2024, Azerbaijan hosted the last COP (29th)—UN Climate Change Conference Baku—where it was agreed to triple finance for developing countries by 2035 [5]. This new agreement will lead to cheaper and cleaner energy, protecting billions of lives from the impacts of climate change, if all nations in the agreement pay their part [5].

What does this mean for society? Addressing climate change entails new ways of living and working. It challenges all governmental institutions as well as private sectors to commit themselves to adaptation and mitigation strategies towards carbon reduction by 2030 and net zero by 2050 [4].

Considering the UN leads policymaking, health providers and the nursing profession should adopt the UN Sustainable Development Goals (SDGs) as a framework to ensure resilient health systems are in place and communities are well prepared to face the challenges brought by climate change. SDGs 6 and 7 (clean water and sanitation; affordable and clean energy) as well as SDGs 11–15 (sustainable cities and communities, responsible consumption and production, climate action, life below water, life on land) are all planetary health-centred and a focus for nursing to act upon [6].

Nurses are amongst the most trusted professions in the world and thus are in a very advantageous position to climate-educate and promote environmental change [7]. The question is: What is nurses’ role in practice, policymaking, advocacy, and education to reduce the health risks from the impacts of climate change?

Since Florence Nightingale introduced the Environmental Theory, nurses have increasingly played a key role in communicating environmental risks [7] as well as presenting climate action to patients, families, and their communities. Since nurses act as key players in educating the public in general, they can also be a resource to their peers on climate literacy and strategies to help the health sector reduce its emissions and control climate health [8].

Nurses are both individuals—managing the consequences of climate change themselves—and professionals, often contributing to climate change in a negative way through healthcare and their actions at the bedside or towards their community, whether intentionally or unintentionally [7].

Reducing healthcare greenhouse gas emissions, implementing strategies for climate efficiency in healthcare facilities, ensuring climate preparedness but also adopting better lifestyles are actions that can be introduced by nurses as individuals but also as a professional group by empowering their peers and communities. As frontline workers fighting climate change, directly or indirectly, nurses can be seen working in catastrophic disasters, pandemics, floods, hurricanes, amongst others. Their role is essential in weather disasters, emergency departments, health centres, and the community by preventing the impacts of climate-related incidents [8].

As an example, since the 2020 pandemic, the healthcare sector has increased plastic waste (by overusing Personal Protective Equipment—PPE), which was one of the COVID-19 pandemic’s environmental side effects [9]. The sector needs to better manage its current carbon footprint by involving all workers—healthcare professionals, managers and leaders, especially those in nursing—to deal with increased energy use [8].

Nurses across the world are not only qualified professionals to lead climate action, but also influencers and educators that can empower colleagues, student nurses, and communities by actively engaging individuals in sustainable climate mitigating actions. This is possible in their four domains: education, practice, research, and policy [10]. As a result, they can intervene to improve community resilience, offering opportunities for citizens’ participation and empowerment to address climate change [11].

Currently, good practices require nurses to identify and diagnose environmental hazards resulting from climate change impacts in urban areas and determine adequate interventions that can lead to behavioural changes [7]. These interventions should follow the United Nations’ guidelines [7] for climate action within cities, such as helping individuals and families to mitigate their negative environmental impact, implementing decarbonizing lifestyles, or adapting to climate impacts. Some of these actions include increasing resilience, reclaiming green spaces, and areas that can provide a positive mental health boost in cities, while also reducing health risks associated with climate change [12]. Additionally, nurses are well positioned to be involved as key stakeholders with the “*medical, public health, and emergency management community in all aspects of disaster planning, mitigation, response, and recovery*” [13].

As to the responsible consumption and production targeted by UN SDG 12, nurses can play a pivotal role by simply asking the questions, “*Can we use less? Can we reuse? Can we recycle?*” [7], and can influence others to ask themselves the same questions. Human diet is the result of consumption habits that have been influenced through the years by historical, cultural, and social influences. The different agrifood systems developed worldwide have adapted to the particularities of each territory, resulting in a diversity of diets around the world. Nevertheless, fossil fuels and pesticides have globally transformed traditional agriculture [14], also resulting in changes in methods of production, distribution, and commercialization around the world. Consequently, this caused diet patterns based on unsustainable practices that impact our planet, leading to higher levels of greenhouse gases (GHGs). Based on data from the Intergovernmental Panel on Climate Change (IPCC) [15], twenty five percent of GHGs originated from agriculture, forestry, and soil use. Hence it is urgent to promote local and regional alternatives and educate populations into decarbonized and sustainable diets [16].

The Final Report of the High-Level Panel of the European Decarbonisation Pathways Initiative, a package of measures to decarbonize lifestyles, developed by the European Commission (EC) in 2018, supports alternative diets and a transition from a beef-based diet to a plant-based, which will eventually translate into reducing dairy consumption and animal proteins and consequently cut GHS by around 10% [17].

Nurses can use their voice to promote decarbonized lifestyles, encouraging people to eat locally sourced fruit and vegetables and decrease the impact on the environment, while improving people’s health [7]. Although nurses may find in this an opportunity for climate health-related promotion, they must consider each population’s context. Some families on low income may not be able to make healthier choices over processed food. And some populations living on islands or in regions with little arable land, for instance, may be dependent on imported products due to the lack of agricultural production [7].

The EC Reports’ package of measures, aligned with the Paris Agreement mentioned above, point to the need for reducing Europe’s carbon footprint to neutral levels in 2050, working towards the concept of “livable cities,” where behaviour change strategies and social innovations are required from policymakers, politicians, and private businesses for replication and implementation [18]. Healthcare workers such as nurses can work alongside urban planners to design cities with cycling pathways, more opportunities for walking, and decreasing carbon emissions caused by vehicles [12]. Nurses can develop “green teams” to promote green infrastructures and sustainable designs for climate resilient cities and institutions [7]. Greenways, for instance, originally defined in the Lille Declaration, in 2000, offer communities healthier lives as they promote non-motorized routes, inspiring closer relationships between citizens and simultaneously decreasing pollution levels in cities [19]. Greenway planning resulting in linear vegetated green spaces are growing around the world and include natural, recreational, and cultural resources [20,21]. Developing such infrastructures will encourage their use as health resources and reduce the impact of climate change, increasing communities’ “health status”.

The UN’s framework, in the context of SDG 7—Affordable and clean energy—sustainability principles should be followed by architects, landscape planners, engineers, and builders, working alongside nurses when planning, designing, constructing, or reshaping healthcare facilities to reduce energy, waste, and GHG emissions. By contributing to reduce the *urban island heating effect* near hospitals or health centre areas, nurses can highlight within these teams the need for planting more trees or using sunshades, creating pedestrian and bicycle access between buildings, and avoiding car use inside or near the facilities [8].

Nurses as global citizens may inspire others and lead by example; using bottom-up approaches that prepare the public and communities to best mitigate and adapt to climate change may prevent our planet from warming up to 4.3 °C by the end of the twenty first century. Nursing interventions are therefore vital in preparing resilient communities. Central to this approach is assessing and diagnosing vulnerable and *at-risk* communities. According to Melo, nurses can anticipate communities’ health needs by addressing each one as a nursing care unit, as framed by his Model of Assessment, Intervention and Community Empowerment (MAIEC) [22]. According to this theoretical model, communities can be considered *nursing care units*, and nurses key professionals in enhancing communities’ health status, by assessing, identifying, and diagnosing health risks due to compromised processes, participation, or leadership, respectively, in relation to any phenomenon [22]—such as climate change. As frontline workers within the Public Health sector, nurses are well placed to implement the necessary interventions and help reduce unwanted health risks from climate change impacts in urban areas. However, working with transdisciplinary teams and establishing partnerships with other key stakeholders for climate action, nurses can enhance their influence, monitoring the most vulnerable by assessing health threats (i.e., infectious diseases), health behaviours, mortality, or morbidity rates within communities. These actions can include contact interventions with such communities—i.e., email or phone alerts on how to best deal with extreme heat and specific air pollution events, as well as helping them develop their emergency preparedness plans.

Fortunately, nursing activism has been launched globally, and environmentally concerned nurses are getting together across the world to face health and climate challenges. Health Care without Harm (HCWH), the American Nurses Association (ANA), and the International Council of Nurses (ICN) are examples of institutions that are highlighting the relevance of the role of nurses in supporting mitigation and adaptation strategies by contributing to climate-related national policies and action plans [23].

Hence, it is vital to review evidence on the current interventions developed by nurses to reduce the health risks from climate change impacts in urban areas and determine if the public health sector is following contemporary scientific and international recommendations for addressing the challenge posed by climate change on the health of urban communities.

For this purpose, a preliminary search on PubMed and MEDLINE Complete was conducted in which no current or in-progress systematic reviews or scoping reviews were found that mapped existing evidence on nursing interventions to reduce health risks from climate change impacts, specifically, in urban settings.

Although Sayre, Rhazi, Carpenter, and Hughes [8] partially addressed the topic, they did not provide any mapping of evidence on such interventions in urban areas. Thus, the authors decided to fill this gap by conducing the current scoping review with the objective of mapping published and unpublished research on this emergent field of intervention.

## 2. Review Question

The present scoping review focused on the following question:

- Which interventions are identified in the literature as being led or implemented by nurses to reduce health risks from climate change in urban areas?

## 3. Inclusion Criteria

### 3.1. Participants

The present review has included articles and studies involving nurses who promote community-based interventions using climate mitigation or adaptation strategies towards a reduction in health risks from climate change impacts in urban settings.

### 3.2. Concepts

The combination of the three core concepts “Community Health Nursing”, “Environmental Health”, and “Climate Change” were at the core of this review:

#### 3.2.1. Community Health Nursing

According to the Portuguese Nursing Professional Association “Community Health Nursing” includes both public health and family health nursing [24], focusing on the empowerment of the individual, population, or community’s needs, engaging in activities such as health promotion, active care, or enabling each one in their own rehabilitation process, supporting all until their end of life.

#### 3.2.2. Environment

The WHO concept of “Environment” [25] is closely linked to health promotion and encloses the current package of measures of the European Commission, aligned with the Paris Agreement from 2015 [4]. To achieve what some authors designate as “livable cities” [26,27] a reduction in the carbon footprint to neutral levels in 2050 is needed, with behaviour change strategies and social innovations developed by policymakers, politicians, and private businesses for replication and dissemination.

#### 3.2.3. Climate Change

Nevertheless, to reduce the world’s carbon footprint as planned, stakeholders and the population, in general, require climate health literacy to acknowledge the contemporary concept of “Climate Change”. Within the present review, this concept was based on the definition used by the United Nations [28] which relates climate change directly or indirectly with human activity, trigging a significant impact on the global atmosphere and creating over time the climate variability that is currently being experienced around the world.

### 3.3. Context

This review will focus solely on interventions in urban environments, including hospital settings as communities or nursing care units [22].

Factors such as the “*urban heat island*” effect and a higher vulnerability amongst urban populations, in relation to the extreme effects of climate change—with the increase of CO_2_ emissions in these settings—led to the choice of this particular research context.

### 3.4. Type of Studies

Within the present review, the authors have considered quantitative, qualitative, and mixed-methods studies for inclusion. Published or unpublished texts, opinion papers, and other grey literature such as thesis/dissertations have also been considered alongside relevant sources obtained from references lists and the authors’ professional network.

## 4. Materials and Methods

This review has followed the JBI (Joanna Briggs Institute) methodology for scoping reviews and was carried out in accordance with the Preferred Reporting Items for Systematic Reviews and Meta-Analyses extension for Scoping Reviews (PRISMA-ScR) checklist [29] (please refer to Table S1).

An a priori *protocol* was conducted and published earlier in 2023 where objectives, inclusion criteria, and methods of analysis were clarified with detail in advance. This protocol has already been registered on the OSF registry platform.

### 4.1. Search Strategy

For the purpose of this review, the authors chose a three-step approach for the search strategy as follows:

I—A preliminary search initially conducted once the authors started to work on their a priori protocol [30] was already registered and published in 2023; PubMed, MEDLINE complete, CINAHL Complete, Scopus, Web of Science, SciELO (Scientific Electronic Library Online), BASE (Bielefeld Academic Search Engine), and RCAAP (Repositório Científico de Acesso Aberto de Portugal) were the databases used to identify the most relevant articles on the subject.

II—A thorough and more updated search was recently conducted on the same databases by using applicable keywords, Mesh descriptors, and other search terms in order to retrieve eligible titles and abstracts (please refer to Table S2).

III—As part of the third step of the search strategy, the authors chose to use a purposive method of data collection in qualitative research by screening references lists of the articles retrieved to add further significant studies which would have been missed otherwise (Snowballing Sampling). Of these, only two full-text articles were considered.

#### Information Sources

For reliability purposes, Google Scholar was not used as it only allows a limited number of documents without retrieving all evidence-based documents required.

All databases mentioned in the previous subsection—PubMed, MEDLINE complete, CINAHL Complete, Scopus, Web of Science, SciELO (Scientific Electronic Library Online), BASE (Bielefeld Academic Search Engine), and RCAAP (Repositório Científico de Acesso Aberto de Portugal)—contributed to retrieve both published and unpublished material, as well as grey literature. Finally, references searched by hand were also considered for inclusion.

### 4.2. Study Selection

The authors’ a priori protocol from 2023 included relevant articles published since 2005—the year in which the Kyoto Protocol was approved. However, as so many new policies and approaches have been contemplated since, a more contemporary perspective is now required. Thus, it was decided for the purpose of the present review that only articles and studies from within the last ten years were to be considered for inclusion, reflecting more current and up to date practices. Ultimately, full-text articles between 1 January 2014 and 31 October 2024, written in English or Portuguese, were selected using the inclusion criteria.

**Figure 1:**
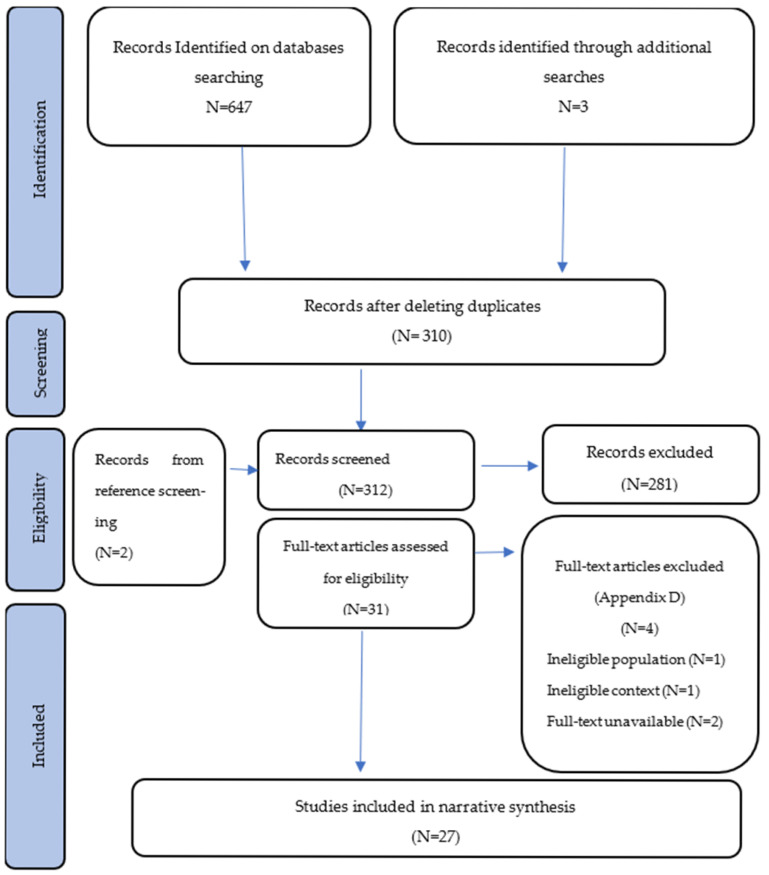
Flow diagram for scoping review process adapted from PRISMA statement by Tricco, Moher, and colleagues [29].

Following a double-blind process performed by two independent reviewers, the search retrieved a total of 650 records. A total of 340 of these records were duplicated studies and consequently deleted, resulting in a total of 310 records to be screened alongside 2 others found using snowballing. From these, an initial review of titles and abstracts was conducted, and 31 full-text articles were initially selected as the most relevant ones. Following thorough and comprehensive review, 4 were excluded (1 due to ineligible population, 1 due to ineligible context, and 2 due to full-text unavailability). In total, 27 were included within the narrative synthesis (please refer to Figure 1—Flow diagram of the review).

Following the database search, all relevant studies identified in the above-mentioned period were uploaded into Rayyan [31]. This platform organizes all data retrieved, identifying duplicated records, resolving each one in a thorough double-blind review process performed by two independent reviewers, against the inclusion criteria.

All disagreements were resolved through discussion, not requiring any third-party involvement.

### 4.3. Data Extraction

A data extraction tool was used by the authors, independently, including detailed information about all interventions identified in the literature as led or implemented by nurses to reduce health risks from climate change in urban areas.

Additionally, the tool developed included information about the authorship of each item included, country of origin, publication type, aim of the study, study design, population, and context.

All disagreements throughout the data extraction stage were resolved through dialogue and academic discussion within the authors.

The data extraction tool is aligned with the aim and objectives of the present review question, with the detailed information for each study item included to support the paper. (please refer to Table A1).

### 4.4. Data Analysis and Presentation

The present review was supported by a data extraction tool as well as a narrative summary of the results encountered, as per recommendations of the JBI scoping review guidelines [32].

By doing so, the authors will describe how each result relates with the aim and question of the review, analysing all data generated through frequency and text analysis, enabling the mapping of the main categories of interventions led or implemented by nurses in urban areas to reduce health risks from climate change.

## 5. Results

### 5.1. Study Inclusion

The database search retrieved a total of 647 studies as well as 3 records identified through additional searches. A total of 340 duplicates were deleted, and 310 records were screened for eligibility alongside 2 additional papers considered using snowballing.

Two independent reviewers using a blind process screened all titles and abstracts, and 31 full-text records were selected for comprehensive reading. Of these, 4 were excluded due to ineligible population (*N* = 1), ineligible context (*N* = 1), or full-text unavailability (*N* = 2) (please refer to File S1).

T 27 records selected for inclusion were exported from Rayyan to a free online version of Zotero, used in the present study as a references management tool, which acted as a facilitator through the selection process.

Both search and selection processes are fully reported in the present study and presented in a Preferred Reporting Items for Systematic Reviews and Meta-Analysis (PRISMA) checklist, clarifying the entire process [29] (please refer to Table S1).

### 5.2. Characteristics of Included Studies

**Figure 2:**
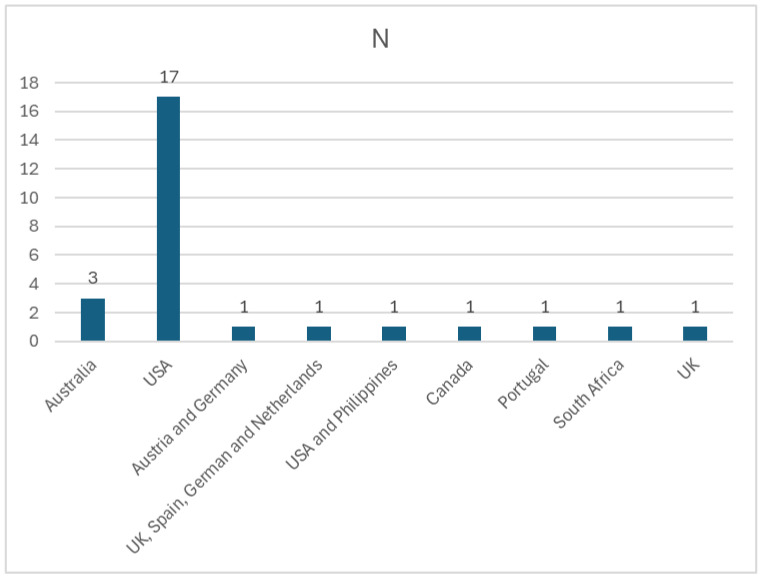
Absolute frequency of geographical location of studies included.

Please refer to Table A1 to locate all studies included and numbered (from 1–27). Most of the studies (eighteen) were developed in the Unites States of America (USA), two of which were conducted in partnership (one with the Philippines). These numbers demonstrate not only the American robust research infrastructure, and large economy, but also its historic emissions and concern regarding climate change impacts on public health. The American Nurses Association (ANA) is an example of an American organization representing millions of registered nurses that has made a valuable and proactive contribution on the dissemination of relevant climate change knowledge, issuing position statements and guidance for professionals [33]. Three studies have been conducted in Australia; one study was conducted in Canada and another one in South Africa. The remaining ones were carried out in Europe—one in Portugal, two in the United Kingdom (UK), one from a partnership between the UK, Spain, Germany, and The Netherlands (please refer to Figure 2).

**Figure 3:**
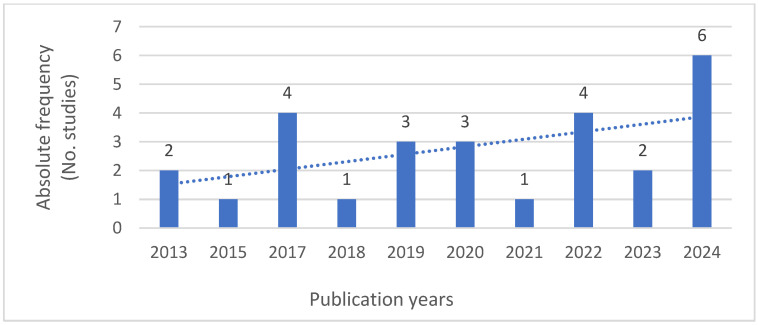
Absolute frequency for publication year of included studies.

As to the year of publication, the authors chose to consider more contemporary studies, between 2014 and 2024, finding twenty-seven relevant studies, two from 2013, one from 2015, four from 2017, one from 2018, three from 2019, three from 2020, one from 2021, four from 2022, two from 2023, and more recently, in 2024, six studies were identified as significant, showing a growing awareness of climate change as a public health issue—please refer to Figure 3.

Amongst the twenty seven papers, the authors identified two case studies (2,21), one Delphi panel used as a study design method (23), one descriptive study (4), one historical research paper (11), two papers using a methodological design format (5,7), four narrative reviews (13,15,16,19), one observational cross-sectional study (10), one recommendations review (9), eight review articles (1,3,6,8,12,17,24,27), three scoping reviews (14,18,20), and three systematic reviews (22,25,26).

**Figure 4:**
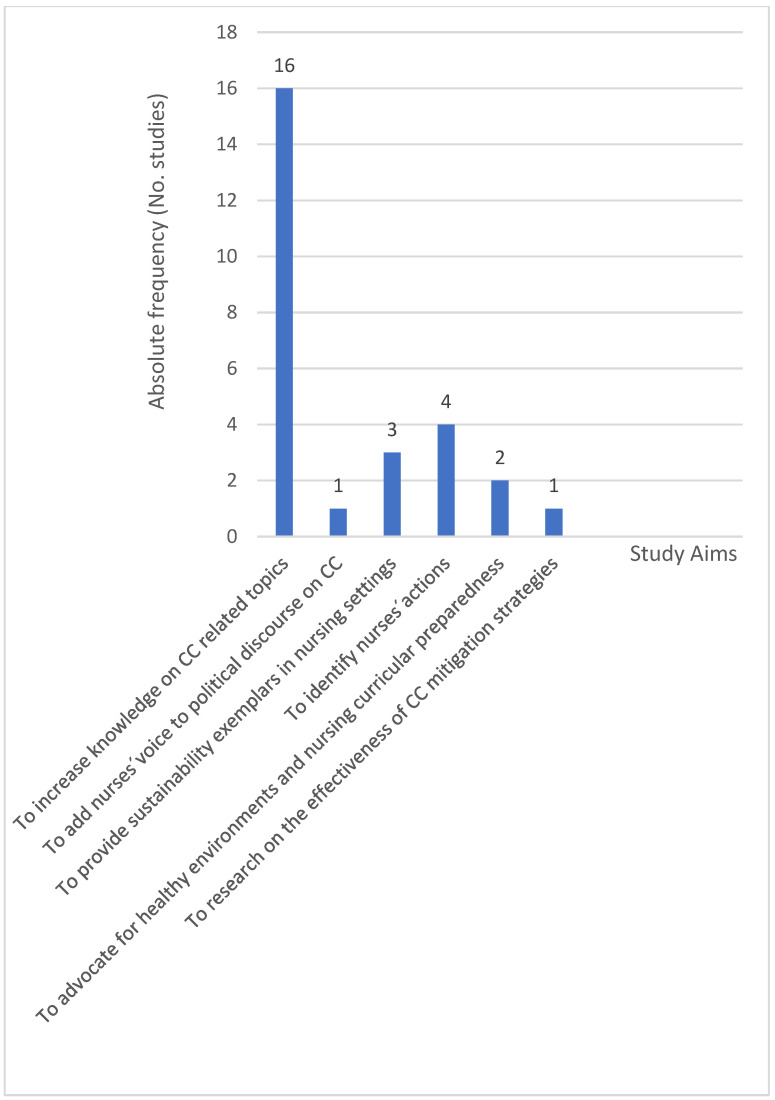
Absolute frequency for aims observed in studies included.

The main aim of sixteen papers was to share or increase the level of knowledge on climate change-related topics (1–7,13,14,16–18,20,24,25,27), followed by four papers whose objective was to identify nurses’ contributions, actions, and solutions to address environmental challenges and to mitigate or adapt to CC (11,12,19,22). Three papers aimed to provide for and evaluate sustainability exemplars in nursing settings (9,10,21), two focused on advocating for healthy learning environments and disaster nursing education, with a focus on nursing curricular preparedness (15,23), one aimed to add nurses‘ voices to the public and political discourse on CC (8), and one (26) aimed to investigate the effectiveness of CC mitigation strategies—please refer to Figure 4.

### 5.3. Review Findings

#### 5.3.1. Population

**Figure 5:**
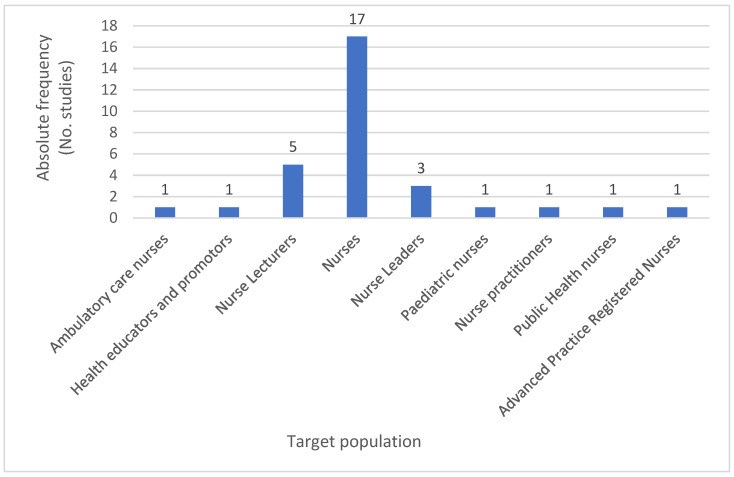
Absolute frequency of population targeted by studies included.

As confirmed by Figure 5 presented below, the authors identified seventeen papers (1,7,11–13,15–25,27) that refer to nurses in general, with five studies (1,4,9,10,27) directed towards nurse lecturers and nurse educators and three to nurse leaders or coordinators (1,14,23), whilst all other papers targeted a population of ambulatory care nurses (2), health educators and promotors (26), pediatric nurses (8), public health nurses (5), or other advanced practice registered nurses (6). It can be inferred that although 100% of the studies targeted nurses, a vast majority (63%) focused on general nurses as a professional group who may have a voice and a role in reducing health risks from climate change, regardless of their area of expertise.

However, the authors wish to highlight that five (18.5%) out of the twenty-seven papers intended to target their content directly to nurse lecturers, educators, and academics. These are key stakeholders with the potential to use their role in society to disseminate CC-related knowledge and literacy amongst students and the new generations of nurses as professionals.

#### 5.3.2. Context

Papers eligible—as per the inclusion criteria requirements presented in a previously published scoping protocol—involved urban contexts and urban communities (such as hospital settings seen as community nursing care units), as these represent a major risk in the health of the urban population due to phenomena such as the “*urban heat island*” factor. Papers targeting exclusively rural contexts were excluded from this study.

As analyzed by the authors, nineteen out of the twenty-seven papers focused on all settings, irrespective of their context, but the remainder of them, eight studies, were exclusively directed to urban studies (4–6,9,10,18,21,27).

#### 5.3.3. Review Question

Within the literature review, the authors defined eight “categories a posteriori” as conceptualized by Bardin [34], enabling a synthesis of the interventions being currently led or implemented by nurses to reduce health risks from climate change in urban areas (please refer to Table 1).

1. *Education and Training interventions* are discussed as actions being implemented by nurses in 24 of the papers (1,2,4,7–27), meaning that 89% of the studies listed the importance of disseminating knowledge on CC amongst nurses, but also on the health consequences associated with it. Educating self, peers, and communities on CC-related diseases as well as building environmental health literacy and empowerment is key for these authors. In this category, a variety of interventions are highlighted as essential for educating and training:
  - Increasing awareness of heat-related illnesses; increasing awareness of evidence-based-practice; increasing awareness of environmental sustainability in nursing practice; increasing awareness of weather related morbidity and mortality (drowning, electrocution, cardiovascular events, and mental health effects); increasing awareness of waterborne diseases (humidity levels, severity of rain, water contamination with pathogens and chemicals); increasing awareness of vector-borne (mosquito and ticks) and zoonotic diseases and symptoms; increasing awareness of recreational water-related illnesses.
  - Increasing knowledge of the mental health impacts (MH) of flooding, displacement, and others.
  - Including/enhancing CC in the educational curriculum in nursing schools at undergraduate and postgraduate levels; increasing student/staff and faculty awareness of CC.
  - Developing case studies or problem-solving teaching; exposing students to disaster training through simulation and exercises; developing electronic tools for nurse educators (simulations, examples, curricular guidelines); promoting active learning about CC amongst students, replacing textbooks with current peer-reviewed publications; promoting “doing something” instead of just passive listening; introducing discussion forum topics and scenario-based learning/mobile learning; providing student feedback to optimize student learning on CC.
  - Offering interprofessional education (social services, etc.).

2. *Clinical interventions* (assessment/screening/prevention/promotion/treatment) are discussed in 16 of the papers included (2,3,5–8,10–12,14–18,20,25), meaning 59% of the studies included autonomous actions from these professionals—eco-nursing knowledge and skills (practicing independently, demonstrating leadership and community engagement) that can be sub-divided in primary, secondary, and tertiary measures as follows:
  - *Primary measures*—preventing CC-related diseases; promoting public and environmental health; preventing children/students in the communities from being exposed to harmful air; educating on prevention measures to reduce indoor air pollution, educating all school-levels teachers on the importance of identifying sources of moisture and mould, opening windows daily, using fans, maintaining ventilation, repairing leaking windows, preventing condensation on cold surfaces; modifying school activities to prevent exposure, practice times or locations; consulting level of outdoor air pollution/smoke/small particles; educating the community about health risks associated with CC; counselling families and their children on the hazards of health pollution; encouraging reduction in activity and remaining indoors during adverse pollution conditions; school nurses to monitor air quality index and students, preventing asthma exacerbation/increasing treatment frequency, etc.; educating families with chronic conditions in preparing for natural disasters or disruption of healthcare with a 1–2 week supply for children; helping to educate families on practices to reduce risk of dermatological diseases; advise parents to keep children off the water in the presence of warning signs of animal droppings, snails, or others; teaching on washing hands/body with clean water and soap afterwards for precautionary measures; teaching on main symptoms (cramps and diarrhea) from contaminated lakes, ponds, swimming pools, etc.; preventing the use of ice made from untreated water; alerting for the presence of Legionella contamination in warm environments; supporting safe water practices; supporting healthier lifestyle choices; promoting new work practices to prevent sedentarism; promoting exercise; educating the population on extreme weather events; raising awareness for CC; disseminating knowledge on emergency preparedness and response, including pediatric population and individuals with special care needs; understanding disaster risk preparedness for effective responses; optimizing health and abilities, preventing weather-related injuries; promoting behaviour change to influence healthier diet choices, by reducing red and processed meat consumption, increasing plant or meat-free alternatives as they can reduce up to 30% of GHG emissions whilst they also reduce the risk of chronic diseases.
  - *Secondary measures*—monitoring vulnerable populations; screening for illnesses; screening for heat intolerance; identifying/diagnosing students at high risk of developing allergy or asthma-related symptoms; identifying diagnosed student–athletes and ensuring appropriate playing time and exertion levels are established; reporting and supporting surveillance of water-related illnesses; assessing heat vulnerability in homebound populations; ensuring mosquito surveillance; ensuring surveillance of endemic and emerging diseases and checking the need for individualized physical and MH care in vulnerable populations.
  - *Tertiary measures*—managing CC-related diseases; assisting students in managing their allergies and asthma, preventing flare-ups, minimizing the requirement of maintenance medication or hospital admissions; developing action plans for each one of those students; ensuring interprofessional collaboration to ensure increase in impact for school nurse roles; early identifying families and children at risk, providing anticipatory care to them; applying suitable communication skills methodologies to promote self-protective behaviour during natural hazards.

3. *Community actions to improve urban resilience* are mentioned in 16 of the papers included (1,6–8,11,12,14–20,22,24,25), meaning that 59% of the studies included refer to local and also organizational actions not only to mitigate carbon emissions but also adapting to the impact already caused by CC:
  - *Local actions—*liaising with local emergency responders so they have a list of people with complex health conditions in the case of extreme weather disasters; offering cooling stations in case of heat waves; engaging with other key stakeholders and having warning and observation systems in place, encouraging children to play outdoors when safe; establishing local recovery groups and enlisting the aid of community networks to mitigate physical/psychological damage.
  - *Organizational actions—*sharing public health announcements on signs and symptoms of heat stress/need for increasing fluid consumption and reduction in activity; engaging with peers in analyzing sub-district vulnerabilities; planning emergency preparedness; increasing the outreach on the concept of sustainability regarding energy efficiency, reducing waste, recycling techniques, awareness of locations for bins for recyclable products (coffee pods, Styrofoam, plastic straws, disposable cutlery, plastic bottles), turning off lights, using hand sanitizer, limiting water usage as well as single use devices (SUDs), using instead alternative disposable products as biodegradable plastics or compostable items (i.e., bamboo cutlery); and ensuring waste is reduced and appropriately diverted as per policy recommendations (recycling effort on paper, metals, sharps, containers, fluorescent lamps, batteries, electronics, organic food waste, pallets, unused medical supplies, and even SUDs); improving health systems resilience and implementing disaster plans for hospitals, assessing flood disaster strategies and social inequalities.

4. *Health policy formulation* is mentioned in 14 (5,6,8,9,11–14,17–20,22,24) of the 27 papers included, meaning that 52% of these studies refer to the following interventions: implementing regulatory programs to reduce GHEs; implementing landfill diversion programs; ensuring proper signage and education are in place; engaging with policymakers; implementing healthcare leadership and role modelling; motivate nurses to follow guidelines for biological/hazardous material disposal; ensuring the reduction of over-prescriptions, ordering smaller dose vials, and educating on pharmaceutical waste; tracking and reporting back on key performance indicators (KPIs), identifying facility champions; sharpening documentation of risk exposures on traffic, crowding, air or water; supporting the creation of an interprofessional collaborative taskforce to prepare care plans for individuals and communities affected by climate-related health problems; ensuring social justice and contributing to the development of health policies.
5. *Environmental Advocacy and Activism interventions* are referred in 13 of the 27 papers included (1,2,4,6,8,9,11,12,15,19,20,22,25), meaning 48% of these studies refer to a variety of actions based on the following: advocating for environmental changes to reduce heat exposure or improve community resilience; leveraging national sustainability initiatives; limiting further environmental degradation and remediating the impacts of CC; advocating to improve urban planning and the development of urban spaces—such as addressing the impact of rising temperatures, applying legal limits on emissions near playgrounds and schools, preventing exposure to air pollutants, etc.; advocating for improved public health policies in place in order to control GHSs and addressing CC consequences on children’s health outcomes; collaborating with other organizations whenever necessary; advocating and linking for composting landscape waste and recycling in municipal and organizational facilities; advocating for regulatory protection and enforcement of international directives and guidelines; promoting dialogue and collaboration between all stakeholders; strengthening the voice of vulnerable populations; supporting automobile fuel economy standards; advocating for policies towards the reduction of carbon-based energy, promoting renewable, clean sources of energy generation in universities and healthcare systems; in regard to outdoor pollution, supporting regulations for 350 ppm or less carbon in the local atmosphere; building a culture of awareness and responsibility around reducing personal contribution to increased atmospheric carbon; advocating the care of all individuals, families, groups, communities, and populations.
6. *Participation in social media/use of digital technology* is quoted in 7 of the papers included (4,8,12,13,17,18,23), meaning that 26% highlight the relevance of nurses in engaging with the public, sharing presentations within their communities, marketing the benefits of behaviour change such as the increase in physical activity and others. Social media is used for public engagement, as are other digital technologies such as distance learning platforms, online courses, virtual simulation, team-based learning, gaming, short message service (sms) messages, news stories, posters, among others.
7. *Green Business interventions* are implied in 8 (1,4,6,9,17,19,21,22) of the 27 papers included, meaning that over 30% of the studies revealed that nurses’ organizational involvement in environmental sustainability is a current trend in clinical and academic settings. Green practices reviewed include the following: promoting energy efficiency, conservation, and sustainability within healthcare settings (i.e., lighting, heating and cooling systems); implementing clear signage and sustainable orientation in laboratories; implementing reuse and recycling programs, resource conservation and the adoption of renewable energy, using waste reduction and net-zero strategies (i.e., electronic medical records, avoiding incorrect disposal of medicines/drugs down the drain, single use plastic segregation and recycling; using interactive resources such as digital education material in academic settings); starting green teams for energy conservation, promoting cycling and walking or organizing bike rides; promoting environmentally friendly actions such as recycling, donating used furniture and using reusable gowns, turn sheets, containers and liners, among others. These greening business operations, climate-friendly practices aim at minimizing waste (i.e., clothing swaps, changing purchasing behaviour, reduction in single-use products, investment in reusable products); reinforcing knowledge on the relation between nursing waste reduction and GHGs reduction); and promoting sustainable purchasing; developing skills and infrastructure within institutions.
8. *Research interventions* are also referred to in 5 (5,12,17,20,22) of the 27 papers included, meaning that 19% of the studies noted nurses’ contributions to preparing articles for peer-reviewed journals or conducting other-related research with climate change and nursing practice and education. In these, some highlight the importance of studies related to the specific needs of children with asthma, to the need for more evidence-based practices, implementing mitigating strategies and working in ways that can lead to proficiency in the climate change domain. Furthermore, one of the studies [26] stressed the importance of supporting nursing research on electronic health records as a best practice for meeting waste reduction requirements for a net-zero healthcare sector.

**Table 1:** Absolute frequency and relative frequency for nursing interventions.

Nursing Interventions	Absolute Frequency (FI)	Relative Frequency (f/n)
Education/Training interventions	24	0.888888889
Clinical interventions	16	0.592592593
Community interventions to improve urban resilience	16	0.592592593
Health policy formulation	14	0.518518519
Environmental advocacy and activism	13	0.481481481
Participation in social media/use of technology	7	0.259259259
Green business interventions	8	0.296296296
Research interventions	5	0.185185185
Total	27	1

## 6. Discussion

The present review comprehensively searched literature between January 2014 and October 2024, retrieving a total of 650 papers, identifying from these 27 relevant studies for inclusion. Within these studies, the authors were able to define eight categories of nursing interventions to reduce health risks from CC in urban areas. The number of studies, although high, reflects a trend towards more discussion of some of the concepts. However, when explored together, the concepts of community health nursing, environmental health, and climate change only revealed a small amount of research, which supports the rationale for the present review. However, through the years, the authors have noticed an increase in interest for researching this particular field to address the health impacts of climate change in our communities and across the globe.

The interventions synthesised above are listed as an evidence-based practice that is currently being implemented to mitigate the health consequences of CC.

Although community health nurses tend to work in the community, they may also work in other settings, along with school nurses, paediatric nurses, mental health nurses, etc. Thus, all nursing professionals working in the community or even in hospitals’ “nursing care units,” a type of small community, have a variety of reasons to engage in CC mitigation and adaptation to reduce health risks on individuals, families, or urban communities.

**Figure 6:**
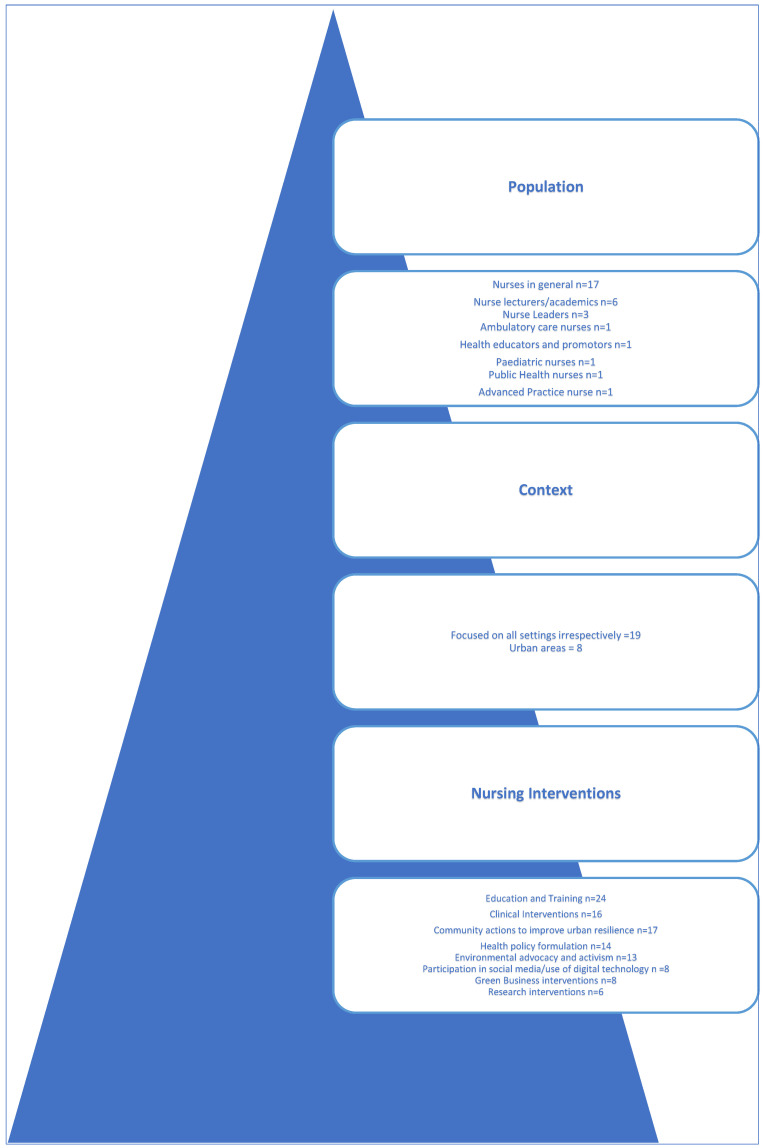
Summary evidence: overview of identified populations with corresponding contexts and type of interventions identified in urban areas.

To revisit the review findings, please refer to Figure 6 below.

The present study reinforces the role of nurses in reducing health risks from climate change by leading or implementing interventions such as the ones described above and enhanced through developing partnerships with other fields.

Authors like Adlong and Dietsch [35] discuss the importance of disseminating education and knowledge about the consequences of climate change. Through this strategy, other peers can be updated with new knowledge and evidence for best practices that will enable them to empower their communities to cope with CC impact.

Sibindi, Chipps, and Crowley [36] highlight the need for diagnosing, managing, and preventing climate-related illnesses. Monitoring vulnerable populations is key to nursing in urban communities that are often subject to stronger impacts from adverse weather events, such as floods. Having the necessary eco-nursing skills is crucial to identify distinct disease burdens, depending on the geographical location of the population. Although professional nurses may be ill-prepared to participate more actively in CC-related initiatives, knowledge deficiencies can be overcome by integrating CC and sustainability topics into nursing curricula and using evidence-based research to improve practice.

However, Bernhardt, Quinn, and Cox [37] stress that there are already innovative strategies being implemented in the field, where CC-related knowledge and evidence-based practices are being applied. This includes the use of screening tools for climate-related illnesses, such as the ones caused by heat waves, which particularly impact older adults. By applying best practices, ambulatory care nurses are now able to identify heat intolerance in particular populations, enabling a reduction in emergency department visits as well as hospital admissions. These practices need to be shared across the professional community.

The present review also shows that nursing responsibilities and interventions continue far beyond clinical care. Veenema, Rush, DePriest, and McCauley [13] discuss the long-standing role that nurses play in ensuring social justice by promoting policy change through new health policies, at different levels, from their places of work, and in close collaboration with other key stakeholders. Disaster planning policies are just one example of these types of proactive policy outcomes.

Authors like McCauley and Hayes reinforce this notion that nurses have always advocated for environmental health since the beginning of the profession, regardless of their specialty or practice setting. They highlight the importance of being involved in environmental activism, protesting against harmful community exposure to environmental impacts, whether they relate to chemicals, environmental hazards, playing behaviours, or time spent in institutional settings that may not adhere to safety standards [38].

According to Radu [39], Wadi, Cheikh, Keung, and Green though, prevention is not a sufficient strategy to address climate change. Societies around the world need to promote new behaviours. Nurses can play a vital role in stressing the importance of engaging in more sustainable dietary behaviours, such as shifting to plant-based diets, which may lead to a 30% reduction on GHG emissions [40]. Social media, marketing, online technology, and platforms are particularly helpful for nurses in clinical settings or nurse lecturers in academic settings to promote the benefits of new dietary behaviours and patterns in societies where the consumption of fruit and vegetables is far below recommendations [40].

In addition to promoting healthier diets, nurses’ public health strategies include advocating for healthier and more sustainable lifestyles such as walking and cycling and promoting exercise and active living for vulnerable groups, according to Salvador Costa, Leitão, Silva, Monteiro, & Melo [12]. Also, other greening strategies may include promoting clothes swapping, waste reduction, water and energy conservation, or developing green teams [39].

To boost green operations in the health sector, data needs to be shared to encourage best practices. Authors such as Sibindi, Chipps, and Crowley [36] and Valentine-Maher, Butterfield, and Laustsen [41] note that proficiency in CC-related nursing research continues to act as a key driver to change practices and implement mitigating strategies by nurses.

## 7. Limitations of Study

Although the present study could have been enhanced with the inclusion of articles describing other interventions related to different extreme weather events, such as fine airborne particulate matter or with articles written in languages other than English and Portuguese, the authors believe that they achieved the main objective for the present review by mapping the literature and the state of the art in the field.

Furthermore, adding studies from continents other than Europe, Africa, or North America could have added value to this manuscript. Nevertheless, however limited, the present review has contributed to the conclusions shared below.

## 8. Conclusions

The authors of the present review were able to identify 27 relevant papers that responded to the key question initially presented for the study. Although research in the field has been increasing, the data analysed and extracted show the need for continuous studies to further identify nursing interventions in the field, worldwide. Moreover, further research is required to study the impact and effectiveness of nursing interventions in the field and how these contribute to reduce health risks from climate change impacts.

Nurses working in urban settings represent the population targeted by the present study. Eight categories of nursing interventions were clearly identified and distinguished within the literature as being currently led or implemented by these healthcare professionals across the globe. These include education and training interventions, clinical interventions, community interventions to improve urban resilience, health policy formulation, environmental advocacy and activism, participation in social media, and use of technology, green business and research.

Nurses play a vital role as key stakeholders in addressing climate change; however, transdisciplinary collaboration is required to reduce existing CC-related health risks. Likewise, more needs to be done in the future, namely in continuing professional and academic development, and ensuring that CC-related knowledge and environmental health are included within nursing curricula, guaranteeing best practices for current and future professionals. Only by better preparing nurses in the field will these professionals be fully equipped to fulfil their role in effectively reducing the health risks of climate change impacts in urban populations across the globe.

## Data Availability

For data supporting reported results please contact the authors of this review.

## Supplementary Materials

The following supporting information can be downloaded at: https://www.mdpi.com/article/10.3390/ijerph22081177/s1, Table S1: Preferred Reporting Items for Systematic Reviews and Meta-Analyses Extension for Scoping Reviews (PRISMA-ScR) Checklist Adapted from PRISMA Statement by Tricco, Moher, and Colleagues [29]; Table S2: Search Strategy Search conducted from 2 October to 25 October 2024.; File S1. Ineligible Articles Following Full-Text Review.

## Appendix A

**Table A1:** Data Extraction Tool of Full-Text Included Articles [12,13,35,36,38,39,40,41,42,43,44,45,46,47,48,49,50,51,52,53,54,55,56,57,58,59,60].

N	Author, Year, Title, and DOI ID	Country	Publication Type	Aim of Study	Nursing Interventions Identified	Study Design	Population (Interventions led by…)	Context
**1**	William Adlong and Elaine Dietsch 2013 Nursing and Climate Change: An Emerging Connection DOI: 10.1016/j.colegn.2013.10.003 [35]	Australia	Journal Article	Assist nurses to recognize the health consequences of climate change, to generate and disseminate knowledge about these health consequences, to be active in mitigating emissions locally and within their organizations, and to advocate and have input into policy processes.	Advocacy for emission mitigation and policy input. Education and dissemination of knowledge about health consequences of climate change Local and organizational actions to reduce emissions Promoting energy efficiency and sustainability within healthcare settings	Review and discussion paper, summarizing existing literature and providing recommendations for nursing practice and education	Interventions are led by nurses, including nurse academics, nurse leaders, and health professionals	Not specific because are general recommendations
**2**	Jean M. Bernhardt, Lisa Quinn, Rachel Cox 2024 The Heat-Related Illness Screening Tool: A Case Study for Populations at Risk DOI: 10.62116/NEC.2024.42.2.59 [42]	USA	Journal Article	The study aims to explore the impact of climate change on heat-related illnesses (HRIs) among older adults and to introduce the Heat-Related Intolerance Screening Tool (HIST) as a method for ambulatory care nurses to identify and mitigate the risk of HRIs.	Screening for heat intolerance using the Heat-Related Intolerance Screening Tool (HIST) Educating and training ambulatory care nurses to identify and mitigate heat-related illnesses Providing resources and support to older adults to prevent heat-related illnesses Advocacy for environmental and policy changes to reduce heat exposure and improve community resilience	Case study approach to demonstrate the application of the Heat-Related Intolerance Screening Tool (HIST) in a real-life scenario	Interventions are led by ambulatory care nurses and other healthcare professionals	Ambulatory care nurses (not specific if it is rural or urban)
**3**	Margaret J. Brown, Bradley P. White, Patrice K. Nicholas 2022 Mental Health Impacts of Climate Change: Considerations for Nurse Practitioners DOI: 10.1016/j.nurpra.2021.07.013 [43]	USA	Journal Article	The study aims to provide an overview of the mental health impacts of climate change, particularly for vulnerable populations, and to introduce screening, assessment, and treatment considerations for nurse practitioners (NPs).	The paper discusses the role of nurse practitioners (NPs) in addressing mental health impacts of climate change, particularly for vulnerable populations. It emphasizes the importance of screening, assessment, and treatment considerations for NPs	Review article that provides an overview of existing literature and theoretical frameworks, particularly Maslow’s Hierarchy of Needs, to discuss the mental health impacts of climate change	Led by nurse practitioners (NPs) and other healthcare providers	Do not specify the context, although it is in mental health services
**4**	Patricia Butterfield, Elizabeth Schenk, Phyllis Eide, Laura Hahn, Julie Postma, Cynthia Fitzgerald, Gail Oneal 2013 Implementing AACN’s Recommendations for Environmental Sustainability in Colleges of Nursing: From Concept to Impact DOI: 10.1016/j.profnurs.2013.10.002 [44]	USA	Journal article	The study aims to describe the implementation of the American Association of Colleges of Nursing (AACN) recommendations for environmental sustainability in a college of nursing. It outlines the steps taken to translate these recommendations into specific actions within the college.	The paper discusses several interventions, including increasing student and faculty awareness, greening business operations, participating in media events, leveraging national sustainability initiatives, and enhancing curricula at undergraduate and postgraduate levels	Descriptive study that outlines the implementation plan and actions taken by the college to promote environmental sustainability	Interventions are led by faculty, staff, and students at the Washington State University College of Nursing	Urban University College of Nursing
**5**	Doede, A.L; Davis, R; DeGuzman, P.B 2020 Use of Trajectory Models to Track Air Pollution from Source to Exposure: A Methodological Approach for Identifying Communities at Risk DOI: 10.1111/phn.12859 [45]	Imperial County, California, United States	Journal Article	To demonstrate the use of air trajectory analysis methods to identify large-scale impacts of airborne pollution on the health of a specific community.	Health policy formulation; research; clinical	Methodological study design	Nurses	Urban setting
**6**	Fuller, MG, Cavanaugh, N, Green, S, Duderstadt, K 2022 Climate Change and State of the Science for Children’s Health and Environmental Health Equity DOI 10.1016/j.pedhc.2021.08.003 [46]	USA	Article in Press	To update the state of the science for healthcare providers and advanced practice registered nurses.	Advocacy with equity lens to limit further environmental degradation and remediate the impacts of climate change Advocacy to improved urban planning and the development of urban spaces (addresses the impact of rising temperatures and limits on emissions near playgrounds and schools, preventing exposure to pollutants Implementation of regulatory program to reduce greenhouse gas emissions Emergency preparation to for climate related natural disasters—including planning for the special pediatric populations with special care needs Climate-friendly practices Minimize waste Access to nutritious foods for children nourishment Primary, secondary, and tertiary measures and early identification of children and families at risk Advocacy for improved Public Health policies to address the consequences of CC on child health outcomes	Review article	Nurses	All settings
**7**	Grothmann, T, Leitner, M, Glas, N, Prutsch, A 2017 A Five-Steps Methodology to Design Communication Formats That Can Contribute to Behaviour Change: The Example of Communication for Health-Protective Behaviour Among Elderly During Heat Waves DOI: 10.1177/2158244017692014 [47]	Austria Germany	Journal Article	Aims at presenting the *use* (not a test, comparison, or improvement) of psychological knowledge and theories (such as PMT, HBM, NAT) for the design of communication measures that have the potential to contribute to behaviour change. (Project) To develop communication formats that have the potential to be more effective than previous communicative public health interventions during heat waves. Specific aims of target group analyses on perceptions that influence behaviour to develop effective communication formats that are able to change these influential perceptions, (b) present the benefits of using scientific psychological knowledge for the design of communication formats, and (c) evaluate communication formats prior to their broad dissemination.	A five-step methodology for designing communication formats that support the motivation of protective behaviour in various risk domains (e.g., climate change, natural hazards) and applied it to health-protective behaviour during heat extremes in Austria: Communication measures: 1—Target group selection 2—Target group analysis 3—Development of target group communication formats 4—Pretest of formats in focus groups 5—Improvement of formats based on pretest results - Nurses can directly reduce the vulnerability of the elderly - Nurses have regular personal contact with elders and relatives and know their medical conditions, tailoring communication to each one in particular. - They are also in a good position to motivate self-protective behaviour of the elderly as face-to-face forms of communication are more effective	Methodological study	Nurses	All settings
**8**	Jackson Allen, Patricia 2015 Primary Care Approaches. Climate Change: It’s Our Problem. *Paediatr. Nurs*, *41*(1), 42–46. [48]	USA	Journal Article	To add nurses voice to the public and political discourse on CC.	- Nurses need to be actively involved in promoting the public health benefits of controlling GHGs and planning for the health consequences of climate change - To be aware of the health consequences of climate change - To provide anticipatory care to children and families - Educate the community about health risks associated with CC - Lobby for Public Health Planning to anticipated health consequence - Counselling children and families on the hazards of air pollution, encouraging to reduce activity and remain inside - School nurses to monitor air quality index as well as students’ activity and symptoms - Children to be instructed to prevent asthma exacerbation by increasing treatment frequency - Educate families with chronic health conditions on planning for disasters and disruption of health care services - Children to have supplies for at least 1–2 weeks - Local emergency responders to have a list of people with complex health conditions in case of extreme weather events - Offer cooling stations in case of heat waves - Public health announcements, signs and symptoms of heat stress, limiting activity and increasing fluid consumption - Expand knowledge on vector-borne diseases (mosquitos and ticks)—symptoms	Review article	Pediatric nurses	All settings
**9**	Levett-Jones, T., Bonnamy, J., Fields, L., Maguire, J., OAM, T.M., Pich, J., Sheridan, L., Lokmic-Tomkins, Z. 2024 Promoting Sustainability in Nursing and Midwifery Clinical Laboratories: Strategies for Resource Reduction, Reuse, and Recycling DOI: 10.1016/j.nedt.2024.106105 [49]	Australia	Journal Article	To provide a series of exemplars that illustrate sustainability initiatives used in four university-based clinical skills laboratories.	Sustainable practices to be included in clinical laboratories education curriculum (clear signage and sustainable orientation) Consider long term environmental benefits Reuse and recycling programs Resource conservation Renewable energy adoption Waste reduction and net-zero strategies—reduce, reuse, recycle: - Simulated electronic medical records (SDG12 and 15) - Renewable energy in CL (SDG7) - Avoid incorrect disposal of medicines/drugs down the drain (SDG 6 and 14) Single-use plastic segregation and recycling (SDG 12)	Recommendations	Nursing and midwifery educators and students	University-based setting (urban)
**10**	Álvarez-Nieto C, Richardson J, Parra-Anguita G, Linares-Abad M, Huss N, Grande-Gascón ML, Grose J, Huynen M, López-Medina IM 2017 Developing Digital Educational Materials for Nursing and Sustainability: The Results of an Observational Study, Nurse Education Today DOI: 10.1016/j.nedt.2017.10.008 [50]	United Kingdom Spain Germany The Netherlands	Journal Article	To test and evaluate digital educational materials on environmental sustainability and health, in the context of university nursing education in different European countries.	- A clear and esthetic design of Climate-related materials generates reflection on environmental issues related to nursing practice - Improved relationship between theory and concepts of environmental sustainability to nursing practice - Development of case studies or problem-solving activities - Use of interactive resources for the Digital Education Material and between students/teachers - Evaluation of the learning process	Observational cross-sectional design	Nursing degree students Nursing professionals Nursing degree teachers Expert advisors Technical experts in evaluating digital learning materials	University settings (urban)
**11**	McCauley, L., Hayes, R 2021 From Florence to Fossil Fuels: Nursing Has Always Been About Environmental Health. DOI: 10.1016/j.outlook.2021.06.007 [38]	US	Article in Press	To describe the history of nursing environmental science. To describe nurses “important contributions to the US environmental Justice Movement.	Admission questionnaires might reveal key information such as tobacco use and occupation, home or workplace info. Optimizing the living environment for long-term wellness and prevention rather than only focusing on optimizing recovery Activism strategies to protest deadly community exposures. Preparing for epidemics Expanding access to medicines Keeping adolescents safe, such as the most vulnerable, with asthma, lead poisoning and cancers, neurobehavioral disorders Recruit diverse stakeholders and lead change in the environmental field Identify and manage environmental injustice, namely vulnerable and impoverished populations Learn about environmental risks and translate this knowledge to patients, policymakers and peers Lead national policy discussions on the extent of the risk and offering preventive strategies to governing bodies Disaster preparedness (curricular and community)	Historic description	Nurses	All settings
**12**	McDermott-Levy, R., Pennea, E., Moore, C. 2023 Protecting Children’s Health: Asthma and Climate Change DOI: 10.1097/NMC.0000000000000927 [51]	USA	Journal Article	To address the impacts of climate change on children with asthma and nursing adaptation responses.	- Nurses to learn about climate change - Climate implications for nursing practice - Advocate for health protective climate change policies - Rely on scientific knowledge to inform - Practice - Contribute to research to address the needs of children with asthma	Review article	Nurses	All settings
**13**	Morgan, Roslyn Elizabeth 2019 Determined Action to Tackle Health Determinants: A Collaborative Response to the Challenge of Climate Change Mitigation in Practice Settings DOI: 10.1891/1078-4535.25.3.195 [52]	Australia	Journal Article	To recognize some of the barriers facing healthcare professionals. To summarize top-down and bottom-up responses for climate change mitigation.	To understand environmental sustainability To be included in policymaking decisions on the execution of mitigation measures To integrate awareness of climate change into undergraduate and postgraduate education and professional ongoing development To develop webpages providing forum for connection and sharing of relevant resources and tools	Narrative review	Nurses	All settings
**14**	Newman, M, Leochico, CFD 2022 Promoting Disaster Preparedness for Children with Special Healthcare Needs: A Scoping Review DOI: 10.1016/j.joclim.2022.100145 [53]	USA Philippines	Journal Article	To contribute to the literature review. To summarize ways to improve disaster preparedness of families of CSHHCN (Children with special care needs).	Disaster preparedness education Having an emergency kit Eight-item checklist in ER Having a clinic nurse coordinator Having programs and policies to improve social support, self-efficacy, and resilience Emergency planning Training of adult caregivers School-based drills and exercises	Scoping review	Nursing coordinators	All settings
**15**	Oerther, S., Manspeaker, S. 2024 The Role of the School Nurse in Addressing Climate-Associated Illnesses: Air Quality DOI: 10.1177/1942602X231200024 [54]	USA	Journal Article	To increase understanding of how air quality affects the health of school-age children. To provide school nurses with (primary, secondary, and tertiary) prevention strategies for ensuring clean and healthy learning environments.	To advocate for healthy indoor environments To safeguard students when the outside air is unhealthy Primary:—prevention of illness, and to avoid students from being exposed to harmful air -Education and prevention measures to lessen indoor air pollution (because of heavy rain, educate teachers on the importance of identifying sources of moisture and mould growth (opening windows daily, using fans, maintain ventilation, watching for and repair leaking windows, avoiding condensation on cold surfaces. Modifying school activities to prevent illness (or practice times, locations and attire), consulting level of outdoor air pollution/wildfire smoke outdoors Secondary: - Screening for illness - Identifying students at high risk of developing allergy or asthma-related symptoms, not yet diagnosed - Identifying diagnosed student athletes ensuring appropriate playing time and exertion levels are established Tertiary approaches: - To assist students in managing their allergies and asthma, preventing flare-ups, and minimizing the need for maintenance medication Have action plans for each one - Interprofessional collaborative efforts enhances the school nurse impact	Narrative review	Nurses	All school settings, urban included
**16**	Oerther S, Oerther DB 2024 The Role of the School Nurse in Addressing Climate-Associated Illnesses: Water. DOI: 10.1177/1942602X231208711 [55]	USA	Journal Article	To raise awareness of the impact of water-related illnesses have on school-age children. To provide nurses with information on the signs and symptoms of these illnesses and prevention tips.	Identify recreational water-related illnesses Help educate families on practices to identify/reduce risk from this pathway (dermatological): To advise parents to keep their children off the water where warning signs of animal droppings, presence of snails, or other health risks Washing with clean water and soap are recommended precautions Gastrointestinal illnesses—cramps and diarrhea from contaminated lakes, ponds, swimming pools, etc. Prevent the use of ice made from untreated water Respiratory illnesses: Legionella in warm environments (reporting and surveillance of water-related illnesses) Avoid/contain outbreaks at an early stage Continuing education to spot the signs of illnesses associated Support healthy lifestyle choices and manage risks. Support safe water practices	Narrative review	Nurses	All settings
**17**	Radu, Raluca 2020 Understanding Climate Change in Nursing Practice: An Educational Tool for Nurses DOI: 10.14288/1.0388735 [39]	Canada	Lesson Plan	To introduce learners to key aspects of climate change. To inform nurses how they can enact feasible solutions within the field.	Engaging our peers: - Preparing articles for peer-reviewed journals - Giving presentation - Organizing bike rides, clothing swaps - Including climate change into the educational curriculum at our universities - Offering cc-related training - Start a green team (energy conservation) Engaging with our community - Using media/social media to engage the public - Collaborate with other organization - Working within our place of work for mitigation and adaptation policies - Engaging policymakers	Literature review	Nurses	All settings
**18**	Salvador Costa, M.J., Leitão, A., Silva, R., Monteiro, V., Melo, P. 2022 Climate Change Prevention through Community Actions and Empowerment: A Scoping Review DOI: 10.3390/ijerph192214645 [12]	Portugal	Journal Article	Identify type and characteristics of community empowerment actions to address climate change.	Actions based on public health and environmental health (hybrid actions): - Public and environmental health promotion - Implementation of programs - Health-sector leadership - Analyzing sub-district vulnerabilities - Emergency preparedness - Warning and observation systems - Monitoring processes and risk awareness - Promoting cycling and walking - Encouraging children to play - Marketing benefits of physical activity - Promoting new work practices to prevent sedentarism - Promoting exercise - Assessing heat vulnerability in homebound populations - Ensuring mosquito surveillance - Educating population on extreme weather events - Raising awareness for climate change - Ensuring surveillance of endemic and emerging diseases	Scoping review	Key stakeholders such as health professionals (nurses)	Urban settings
**19**	Schenk, Elizabeth C., Johnson, Sarah, Kelley-Gustafson, Brandi, Turley, Oriana 2023 Nursing Waste Reduction for a Healthy Environment. DOI: 10.1016/j.jradnu.2022.12.008 [56]	USA	Journal Article	To identify waste streams and nursing actions towards a waste optimization plan.	Recycling Composting Landfill diversion programs Changing purchasing behaviour (reduction in single use products, investment in products that are reusable) Municipal solid waste—teach others between the MSW and GHG Biohazardous waste—nurses may segregate biohazardous waste, in order to only collect what requires treatment. Proper signage and education are in place Pathological waste, chemotherapy, and sharps are typically incinerated whereas blood is typically autoclaved and landfilled Hazardous waste—nurses to follow guidelines for hazardous material disposal. Pharmaceutical waste—nurses to reduce over-prescribing, order smaller dose vials, educate on pharmaceutical waste Diverted composting—nurses to advocate and link for compost landscape waste. Recycling—nurses can partner with local municipal recycling centres Universal waste—ensure proper segregation Donations—furniture, food, and medical supplies. Reusable items—nurses to advocate for reusable isolation gowns, turn sheets, containers, and linens	Narrative review	Nurses	All settings
**20**	Sibindi, T, Crowley, T 2024 Eco-nursing competencies for nurses: A scoping review DOI: 10.1016/j.ijnsa.2024.100221 [36]	South Africa	Journal Article	To synthesize the existing literature on eco-nursing roles and competencies for nurses.	- Advocacy for environmentally friendly actions and practices - Conduct research related to climate change and change practice or implement mitigation strategies - Educate self and peers and communities on climate-related diseases - Clinical practice—diagnose, manage and prevent climate-related illnesses; monitor vulnerable population - Leadership—leading through role modelling and leading public health policy actions - Eco-nursing knowledge - Eco-nursing skills (practicing independently, demonstrating leadership community engagement and effective advocacy, proficiency in research and evidence-based practice, emergency preparedness and response)	Scoping review	Nurses	All settings
**21**	Stamps, Deborah C., Waller, Michael G., Foley, Susan M., Gales, Jennifer, Alley, Rebecca, Lovetro, Cindy, Opett, Kristin, Glessner, Theresa, Faggiano, Sheri 2020 Chief Nursing Officer Council Partners with Sustainability Department to Develop a Model of Success for Reducing the Organization’s Carbon Footprint. DOI: 10.1016/j.mnl.2020.01.006 [57]	USA	Article Journal	To show how the CNO council of a healthcare system partnered with the sustainability department to integrate sustainability into daily operations.	- Community outreach and education on the concept of sustainability, energy efficiency methods, reducing waste methods, recycling techniques in workspace (location for bins for recyclable products—coffee pods, Styrofoam, plastic straws, disposable cutlery, plastic bottles—turning off lights, using hand sanitizer, limiting water usage, single use device (SUD) reprocessing efforts, and others), proper waste disposal techniques, alternative disposable products, common item alternatives (plastic cutlery, plastic bottles, reusable vs. disposable), hand sanitizer use over soap and water, donation programs, single-use device reprocessing - Built environment - Waste reduction and diversion (paper, metals, sharps, containers, fluorescent lamps, batteries, electronics, organic food waste, pallets, unused medical supplies, and SUDs) - Food sourcing and waste - Sustainable purchasing - Energy systems and conservation (lighting, heating and cooling systems) - 501©3 donation Intervol a nonprofit organization thar reduces carbon footprint - Device reprocessing - Track and report on key performance indicators (KPIs) - Identify facility champions - Development of an educational curriculum for reference (sustainability discipline was added to the nursing curriculum)	Case-study	Nurses	Hospitals, ambulatory surgery centres, medical groups, long-term-care facilities—all settings
**22**	Valentine-Maher, S.K., Butterfield, P.G., Laustsen, G. 2018 Environmental Health Advancing Emancipatory Policies For the Common Good DOI: 10.1097/ANS.0000000000000194 [41]	USA	Journal Article	Aims for nursing action to address the natural environment, health injustices, improving health for all.	- Build environmental health literacy and empowerment - Advocacy for regulatory protection and enforcement - Environmental engagement with healthcare systems (developing skills and infrastructure within institutions—electronic health records: support nursing research) - Support sustainable practices such as sustainable purchasing, energy/water conservation, improved waste management - Support environmental natural settings - Promote dialogue and collaboration between all stakeholders - Strengthen the voice of vulnerable populations - Sharpen documentation of risk exposures on traffic, crowding, air or water pollution - Apply inequality metrics in explaining some of the impact assessments - Address health disparities - Environmental justice - Oppose protective automobile fuel economy standards - Advocate for policies which reduce carbon-based energy and promote renewable, clean sources of energy generation in universities and healthcare systems - Align with 350 ppm or less carbon in the atmosphere - Build culture of awareness and responsibility around reducing personal contribution to increased atmospheric carbon - Support the creation of interprofessional collaborative taskforce to prepare care plans for individuals and communities affected by climate-related health problems or disasters and climate-related events	Systematic review	Nurses	All settings, clinical and community-based
**23**	Veenema, T.G., Lavin, R.P., Griffin, A., Gable, A.R., Couig, M.P., Dobalian, A. 2017 Call to Action: The Case for Advancing Disaster Nursing Education in the United States DOI: 10.1111/jnu.12338 [58]	USA	Journal Article	To articulate a mandate for the advancement of disaster nursing education within the United States.	- School of Nursing (SON)—expose students to disaster training through simulation, exercises; develop an electronic tool for nurse educators (simulations, examples, curricular guidelines); ethical guidance vs. with chain of command; use of social media and technology, distance learning, online courses, virtual simulation, team-based learning, gaming - Interprofessional education - (social services, mental health services)	Delphi panel	Nurses and nurse leaders	All settings
**24**	Veenema, TG, Rush, Z, DePriest, K, McCauley, L 2019 Climate Change-Related Hurricane Impact on Puerto Rico and the US Virgin Islands, Environment Risk Reduction, and the Social Determinants of Health *Nurs. Econ., 37*(1), 13–22 [13]	USA Puerto Rico	Journal Article	To reveal the cascading effects of the 2017 hurricane season on the social determinants of health.	- Understanding disaster risk - Strengthening disaster risk governance - Investing in disaster risk reduction for resilience - Enhancing disaster preparedness for effective response - Promote economic stability (employment, food insecurity, housing instability, and poverty) - Promote education - Social and community context Health and Healthcare: - Nurses’ responsibilities extend far beyond clinical care as they consist in the optimization of health and abilities, prevention of illness and injury, and advocacy in the care of individuals, families, groups, communities, and populations - Ensure social justice and health policy formulation - Engage in disaster planning - Promote disaster risk reduction - Champion for vulnerable populations - Reduce healthcare’s climate footprint (HCWH 2019) - Include Social determinants of Health (SDOH) metrics into planning and provide support for the policies who will impact the most vulnerable	Literature review	Nurses	
**25**	Veenema, T.G., Thornton, C.P., Lavin, R.P., Bender, A.K., Seal, S., Corley, A. 2017 Climate Change–Related Water Disasters’ Impact on Population Health DOI: 10.1111/jnu.12328 [59]	USA	Journal Article	To conduct a systematic review of the literature concerning the impact of CCRWDs on public health in order to identify factors in these events that are amenable to preparedness and mitigation.	CCRWD - Weather-related morbidity and mortality (drowning, electrocution, cardiovascular events and MH effects) - Waterborne diseases (humidity levels, severity of rain, water contamination with pathogens and chemicals) - Vector-borne and zoonotic diseases (relocation and displacement of large human populations) - Mental health: - To monitor the impact of flooding on the health and wellbeing of those displaced, checking the need for mental healthcare among the affected - Establishing local recovery groups and enlisting the aid of community networks to mitigate physical and psychological damage - Improve the resilience of health systems (operating procedures, functional workforce, policy and land use mechanisms, disaster plans for hospitals, assessing flood disaster strategies, and social inequalities awareness)	Systematic review	Nurses	All settings
**26**	Wadi, N.M., Cheikh, K., Keung, Y.W., Green, R. 2024 Investigating Intervention Components and Their Effectiveness in Promoting Environmentally Sustainable Diets: A Systematic Review DOI: 10.1016/S2542-5196(24)00064-0 [40]	UK	Journal Article	To investigate, classify, and assess the effectiveness of interventions that promote environmentally sustainable diets in high-income countries.	- Education presentation, posters, SMS messages, news stories, school or university courses - Educational interventions are the most effective (associated with motivation and capability) - Influence physical capability and self-efficacy to partake in behaviour change regarding guidelines on red and processed meat consumption or improving access to plant and meat-free alternatives, that can reduce up to 30% of GHG emissions from diets and also reduce the risk of chronic disease	Systematic review	Educators, trainers, health educators, health promotors that promote environmentally sustainable diets	All settings
**27**	Wasco, J.J. 2019 Strategies for Teaching Online RN-to-BSN Students the Health Impacts of Climate Change DOI: 10.1891/1078-4535.25.3.e1 [60]	USA	Journal Article	To introduce the impacts of climate change on nursing care delivery and share the pedagogy of an introductory course developed for an online, post-registered RN-BSN program based at a university.	- To promote active learning - Replace textbooks with current peer-reviewed publications - Promote doing something instead of passive listening - Introduce discussion forum topics and scenarios - Mobile learning - Assignments (infographic, public service announcement, slide presentation, or poster) - Having student feedback	Recommendations review	Nurses/nurse lecturers	Universities

## References

[1] Kurth A.E. (2017). Planetary Health and the Role of Nursing: A Call to Action. J. Nurs. Scholarsh..

[2] Costello A., Abbas M., Allen A., Ball S., Bell S., Bellamy R., Friel S., Groce N., Johnson A., Kett M. (2009). Managing the Health Effects of Climate Change: Lancet and University College London Institute for Global Health Commission. Lancet.

[3] United Nations from Stockholm to Kyoto: A Brief History of Climate Change. https://www.un.org/en/chronicle/article/stockholm-kyoto-brief-history-climate-change.

[4] UNFCCC (2015). Paris Agreement to the United Nations Framework Convention on Climate Change.

[5] UN Climate Change Conference Baku—November 2024 | UNFCCC. https://unfccc.int/cop29.

[6] Rosa W.E., Schenk E., Travers J.L., Nicholas P.K. (2019). Climate Change and Health Consequences: Engaging Public Health Nursing within the Framework of the United Nations Sustainable Development Goals. Public Health Nurs..

[7] Lilienfeld E., Nicholas P.K., Breakey S., Corless I.B. (2018). Addressing Climate Change through a Nursing Lens within the Framework of the United Nations Sustainable Development Goals. Nurs. Outlook.

[8] Sayre L., Rhazi N., Carpenter H., Hughes N.L. (2010). Climate Change and Human Health: The Role of Nurses in Confronting the Issue. Nurs. Adm. Q..

[9] Seif F., Noorimotlagh Z., Mirzaee S.A., Kalantar M., Barati B., Fard M.E., Fard N.K. (2021). The SARS-CoV-2 (COVID-19) Pandemic in Hospital: An Insight into Environmental Surfaces Contamination, Disinfectants’ Efficiency, and Estimation of Plastic Waste Production. Environ. Res..

[10] Vold L., Meszaros M. (2021). Rhizomatic Assemblages: Connecting Climate Change to Nursing Action. Witn. Can. J. Crit. Nurs. Discourse.

[11] Zimmerman M.A., Rappaport J., Seidman E. (2000). Empowerment Theory. Handbook of Community Psychology.

[12] Salvador Costa M.J., Leitão A., Silva R., Monteiro V., Melo P. (2022). Climate Change Prevention through Community Actions and Empowerment: A Scoping Review. Int. J. Environ. Res. Public. Health.

[13] Veenema T., Rush Z., DePriest K., McCauley L. (2019). Climate Change-Related Hurricane Impact on Puerto Rico and the U.S. Virgin Islands, Environmental Risk Reduction, and the Social Determinants of Health. Nurs. Econ..

[14] Magliano D.J., Loh V.H.Y., Harding J.L., Botton J., Shaw J.E. (2014). Persistent Organic Pollutants and Diabetes: A Review of the Epidemiological Evidence. Diabetes Metab..

[15] IPCC Contribution of Working Group III to the Fifth Assessment Report of the Intergovernmental Panel on Climate Change (2015). Climate Change 2014: Mitigation of Climate Change.

[16] Carvalho S., Bisquert i Pérez K., Meira Cartea P.Á., Azeiteiro U. (2022). Descarbonizar a Dieta Através Dos Equipamentos Para a Educação Ambiental. A Educación Para o Cambio Climático no Sistema Educativo. ACTAS IV Seminario Internacional Resclima y 2^o^ Encuentro de la REAJA 26 y 27 de Octubre de 2018.

[17] European’s Commission: Directorate-General for Research and Innovation (2018). Final Report of the High-Level Panel of the European Decarbonisation Pathways Initiative..

[18] European Commission Horizon Europe Work Programme 2021–2022 (2022). European Commission Decision C(2022)2975 of 10 May 2022.

[19] Hamin E.M., Abunnasr Y., Ryan R.L. (2019). Planning for Climate Change: A Reader in Green Infrastructure and Sustainable Design for Resilient Cities.

[20] Fábos J.G., Ryan R.L. (2006). An Introduction to Greenway Planning around the World. Landsc. Urban Plan..

[21] The European Greenways Association Greenways. https://www.aevv-egwa.org/awards/.

[22] Melo P. (2020). Enfermagem de Saúde Comunitária e de Saúde Pública [Community and Public Health Nursing].

[23] HCWH Who We Are?. https://noharm-europe.org/content/europe/who-we-are.

[24] Ordem Dos Enfermeiros Especialidades. https://www.ordemenfermeiros.pt/faqs/especialidades/.

[25] World Health Organisation Environmental Health. https://www.who.int/health-topics/environmental-health.

[26] Aboulnaga M., Trombadore A., Mostafa M., Abouaiana A. (2024). Livable Cities: Urban Heat Islands Mitigation for Climate Change Adaptation Through Urban Greening.

[27] Aboulnaga M., Sala M., Trombadore A., Bisello A., Vettorato D., Haarstad H., Borsboom-van Beurden J. (2021). Open Innovation Strategies, Green Policies, and Action Plans for Sustainable Cities—Challenges, Opportunities, and Approaches. International Conference on Smart and Sustainable Planning for Cities and Regions.

[28] United Nations (1992). United Nations Framework Convention on Climate Change 1992.

[29] Tricco A.C., Lillie E., Zarin W., O’Brien K.K., Colquhoun H., Levac D., Moher D., Peters M.D.J., Horsley T., Weeks L. (2018). PRISMA Extension for Scoping Reviews (PRISMA-ScR): Checklist and Explanation. Ann. Intern. Med..

[30] Salvador Costa M.J., Melo P., Azeiteiro U., Carvalho S., Ryan R. (2023). Nursing Interventions to Reduce Health Risks from Climate Change Impact in Urban Areas: A Scoping Review Protocol. Nurs. Rep..

[31] Ouzzani M., Hammady H., Fedorowicz Z., Elmagarmid A. (2016). Rayyan—A Web and Mobile App for Systematic Reviews. Syst. Rev..

[32] Peters M., Godfrey C., McInerney P., Munn Z., Tricco A.C. (2020). Khali Chapter 11: Scoping Reviews. JBI Manual for Evidence Synthesis.

[33] American Nurses Association (2024). ANA Strategic Plan.

[34] Bardin L. (2011). Content Analysis.

[35] Adlong W., Dietsch E. (2015). Nursing and Climate Change: An Emerging Connection. Coll. R. Coll. Nurs. Aust..

[36] Sibindi T., Crowley T. (2024). Eco-Nursing Competencies for Nurses: A Scoping Review. Int. J. Nurs. Stud. Adv..

[37] Bernhardt J.M., Breakey S., Sipe M., Nicholas P.K. (2023). The Future of Nursing 2020–2030: The Critical Role of Nurses and Nurse Leaders in Addressing the Health Impacts of Climate Change. JONA: J. Nurs. Adm..

[38] McCauley L., Hayes R. (2021). From Florence to Fossil Fuels: Nursing Has Always Been about Environmental Health. Nurs. Outlook.

[39] Radu R. (2020). Understanding Climate Change in Nursing Practice: An Educational Tool for Nurses. Bachelor’s Thesis.

[40] Wadi N.M., Cheikh K., Keung Y.W., Green R. (2024). Investigating Intervention Components and Their Effectiveness in Promoting Environmentally Sustainable Diets: A Systematic Review. Lancet Planet. Health.

[41] Valentine-Maher S.K., Butterfield P.G., Laustsen G. (2018). Environmental Health Advancing Emancipatory Policies for the Common Good. Adv. Nurs. Sci..

[42] Bernhardt J.M., Quinn L., Cox R. (2024). The Heat-Related Illness Screening Tool: A Case Study for Populations at Risk. Nurs. Econ..

[43] Brown M.J., White B.P., Nicholas P.K. (2021). Mental Health Impacts of Climate Change: Considerations for Nurse Practitioners. J. Nurse Pr..

[44] Butterfield P., Schenk E., Eide P., Hahn L., Postma J., Fitzgerald C., Oneal G. (2014). Implementing AACN’s Recommendations for Environmental Sustainability in Colleges of Nursing: From Concept to Impact. J. Prof. Nurs..

[45] Doede A.L., Davis R., DeGuzman P.B. (2021). Use of trajectory models to track air pollution from source to exposure: A methodological approach for identifying communities at risk. Public Heal. Nurs..

[46] Fuller M.G., Cavanaugh N., Green S., Duderstadt K. (2022). Climate Change and State of the Science for Children’s Health and Environmental Health Equity. J. Pediatr. Heal. Care.

[47] Grothmann T., Leitner M., Glas N., Prutsch A. (2017). A Five-Steps Methodology to Design Communication Formats That Can Contribute to Behavior Change. SAGE Open.

[48] Jackson Allen P. (2015). Primary Care Approaches. Climate Change: It’s Our Problem. Paediatr. Nurs..

[49] Levett-Jones T., Bonnamy J., Fields L., Maguire J., Oam T.M., Pich J., Sheridan L., Lok-mic-Tomkins Z. (2024). Promoting sustainability in nursing and midwifery clinical laboratories: Strategies for resource reduction, reuse, and recycling. Nurse Educ. Today.

[50] Álvarez-Nieto C., Richardson J., Parra-Anguita G., Linares-Abad M., Huss N., Grande-Gascón M.L., Grose J., Huynen M., López-Medina I.M. (2018). Developing digital educational materials for nursing and sustainability: The results of an observational study. Nurse Educ. Today.

[51] McDermott-Levy R.P., Pennea E.B., Moore C.M. (2023). Protecting Children’s Health. MCN Am. J. Matern. Nurs..

[52] Morgan R.E. (2019). Determined Action to Tackle Health Determinants: A Collaborative Response to the Challenge of Climate Change Mitigation in Practice Settings. Creative Nurs..

[53] Newman M., Leochico C.F.D. (2022). Promoting disaster preparedness for children with special healthcare needs: A scoping review. J. Clim. Chang. Heal..

[54] Oerther S., Manspeaker S. (2023). The Role of the School Nurse in Addressing Climate-Associated Ill-nesses: Air Quality. NASN Sch. Nurse.

[55] Oerther S., Oerther D.B. (2023). The Role of the School Nurse in Addressing Climate-Associated Ill-nesses: Water. NASN Sch. Nurse.

[56] Schenk E.C., Johnson S., Kelley-Gustafson B., Turley O. (2023). Nursing Waste Reduction for a Healthy Environment. J. Radiol. Nurs..

[57] Stamps D.C., Waller M.G., Foley S.M., Gales J., Alley R., Lovetro C., Opett K., Glessner T., Faggiano S. (2020). Chief Nursing Officer Council Partners with Sustainability Department to Develop a Model of Success for Reducing the Organization’s Carbon Footprint. Nurse Lead..

[58] Veenema T.G., Lavin R.P., Griffin A., Gable A.R., Couig M.P., Dobalian A. (2017). Call to Action: The Case for Advancing Disaster Nursing Education in the United States. J. Nurs. Sch..

[59] Veenema T.G., Thornton C.P., Lavin R.P., Bender A.K., Seal S., Corley A. (2017). Climate Change–Related Water Disasters’ Impact on Population Health. J. Nurs. Sch..

[60] Wasco J.J. (2019). Strategies for Teaching Online RN-to-BSN Students the Health Impacts of Climate Change. Creative Nurs..

