# Supplementary material for: Nursing Interventions to Reduce Health Risks from Climate Change Impact in Urban Areas: A Scoping Review
Source: Int J Environ Res Public Health. 2025 Jul 25;22(8):1177. doi: 10.3390/ijerph22081177 (PMC12386565; doi:10.3390/ijerph22081177)
Supplement: Supplementary file 1 [file ijerph-22-01177-s001.zip › ijerph-3703659-supplementary.pdf]

Table S1. Preferred Reporting Items for Systematic Reviews and Meta-Analyses Extension for Scoping Reviews (PRISMA-ScR)
Checklist Adapted from PRISMA Statement by Tricco, Moher, and
Colleagues [29]

Section	Item	PRISMA-ScR Checklist Item	Reported on Page #
**Title**			
Title	1	Identify the report as a scoping review.	1
**Abstract**			
Structured summary	2	Provide a structured summary that includes (as applicable): background, objectives, eligibility criteria, sources of evidence, charting methods, results, and conclusions that relate to the review questions and objectives.	1
**Introduction**			
Rationale	3	Describe the rationale for the review in the context of what is already known. Explain why the review questions/objectives lend themselves to a scoping review approach.	2
Objectives	4	Provide an explicit statement of the questions and objectives being addressed with reference to their key elements (e.g., population or participants, concepts, and context) or other relevant key elements used to conceptualize the review questions and/or objectives.	4-5
**Methods**			
Protocol and registration	5	Indicate whether a review protocol exists; state if and where it can be accessed (e.g., a Web address); and if available, provide registration information, including the registration number.	6
Eligibility criteria	6	Specify characteristics of the sources of evidence used as eligibility criteria (e.g., years considered, language, and publication status) and provide a rationale.	5
Information sources *	7	Describe all information sources in the search (e.g., databases with dates of coverage and contact with authors to identify additional sources), as well as the date the most recent search was executed.	6
Search	8	Present the full electronic search strategy for at least 1 database, including any limits used, such that it could be repeated.	6
Selection of sources of evidence †	9	State the process for selecting sources of evidence (i.e., screening and eligibility) included in the scoping review.	7
Data charting process ‡	10	Describe the methods of charting data from the included sources of evidence (e.g., calibrated	7

		forms or forms that have been tested by the team before their use, and whether data charting was done independently or in duplicate) and any processes for obtaining and confirming data from investigators.	
Data items	11	List and define all variables for which data were sought and any assumptions and simplifications made.	7
Critical appraisal of individual sources of evidence §	12	If done, provide a rationale for conducting a critical appraisal of included sources of evidence; describe the methods used and how this information was used in any data synthesis (if appropriate).	N/A
Synthesis of results	13	Describe the methods of handling and summarizing the data that were charted.	8
**Results**			
Selection of sources of evidence	14	Give numbers of sources of evidence screened, assessed for eligibility, and included in the review, with reasons for exclusions at each stage, ideally using a flow diagram.	9
Characteristics of sources of evidence	15	For each source of evidence, present characteristics for which data were charted and provide the citations.	9–11
Critical appraisal within sources of evidence	16	If done, present data on critical appraisal of included sources of evidence (see item 12).	N/A
Results of individual sources of evidence	17	For each included source of evidence, present the relevant data that were charted that relate to the review questions and objectives.	12–17
Synthesis of results	18	Summarize and/or present the charting results as they relate to the review questions and objectives.	17
**Discussion**			
Summary of evidence	19	Summarize the main results (including an overview of concepts, themes, and types of evidence available), link to the review questions and objectives, and consider the relevance to key groups.	17–18
Limitations	20	Discuss the limitations of the scoping review process.	19
Conclusions	21	Provide a general interpretation of the results with respect to the review questions and objectives, as well as potential implications and/or next steps.	20
**Funding**			
Funding	22	Describe sources of funding for the included sources of evidence, as well as sources of funding for the scoping review. Describe the role of the funders of the scoping review.	20

Adapted version from Tricco AC, Lillie E, Zarin W, O'Brien KK, Colquhoun H, Levac D et al. PRISMA Extension for Scoping Reviews (PRISMAScR): Checklist and Explanation. Ann Intern Med. 2018; 169:467–473. doi: 10.7326/M18-0850 [29].

Table S2. Search Strategy

Search conducted from 2 October to 25 October 2024.

Source	Query	Records Retrieved 25 October 2024	Result with Filters: English/Portuguese Date 2014–2024
PubMed	((((“climate change” [Title] OR “global warming” [Title] OR “greenhouse effect” [Title]) OR (“climate change” [MeSH Terms])) OR (“global warming” [MeSH Terms])) AND ((((((((“Preventive Health Service *” [Title] OR “Primary Prevention” [Title] OR empowerment [Title] OR “patient participation” [Title] OR “Consumer Participation” [Title] OR “health promotion” [Title] OR “environmental health” [Title] OR “public health” [Title]) OR (empowerment [MeSH Terms])) OR (“patient participation” [MeSH Terms])) OR (“health promotion” [MeSH Terms])) OR (“environmental health” [MeSH Terms])) OR (“public health” [MeSH Terms])) OR (“Preventive Health Services” [MeSH Terms])) OR (“Primary Prevention” [MeSH Terms]))) AND (((Nursing [Title] OR “community health nursing” [Title]) OR (nursing [MeSH Terms])) OR (“community health nursing” [MeSH Terms]))	60	50
Medline Complete	(TI (“climate change” OR “global warming” OR “greenhouse effect”) OR MH (“climate change” OR “global warming”)) AND (TI (“Preventive Health Service *” OR “Primary Prevention” OR empowerment OR “patient participation” OR “Consumer Participation” OR “health promotion” OR “environmental health” OR “public health”) OR MH (empowerment OR “patient participation” OR “health promotion” OR “environmental health” OR “public health” OR “Preventive Health Services” OR “Primary Prevention”)) AND (TI (Nursing OR “community health nursing”) OR MH (nursing OR “community health nursing”))	23	23
CINAHL Complete	(TI (“climate change” OR “global warming” OR “greenhouse effect”) OR MH (“climate change” OR “greenhouse effect”)) AND (TI (“Preventive Health Service *” OR “Primary Prevention” OR empowerment OR “patient participation” OR “Consumer Participation” OR “health promotion” OR “environmental health” OR “public health”) OR MH (empowerment OR “Consumer Participation” OR “health promotion” OR “environmental health” OR “public health”)) AND (TI (Nursing OR “community health nursing”) OR MH “community health nursing”)	91	85
Scopus	(TITLE-ABS-KEY (“climate change” OR “global warming” OR “greenhouse effect”) AND TITLE-ABS-KEY (“Preventive Health Service *” OR “Primary Prevention” OR empowerment OR “patient participation” OR “Consumer Participation” OR “health promotion” OR “environmental health” OR “public	187	162

	health”) AND TITLE-ABS-KEY (nursing OR “community health nursing”))		
Embase	Sem acesso		
Web of Science	TS = (“climate change” OR “global warming” OR “greenhouse effect”) AND TS = (“Preventive Health Service *” OR “Primary Prevention” OR empowerment OR “patient participation” OR “Consumer Participation” OR “health promotion” OR “environmental health” OR “public health”) AND TS = (Nursing OR “community health nursing”)	151	138
SciELO (through EDS)	(TI (“climate change” OR “global warming” OR “greenhouse effect”) OR AB (“climate change” OR “global warming” OR “greenhouse effect”) OR SU (“climate change” OR “global warming” OR “greenhouse effect”)) AND (TI (“Preventive Health Service *” OR “Primary Prevention” OR empowerment OR “patient participation” OR “Consumer Participation” OR “health promotion” OR “environmental health” OR “public health”) OR AB (“Preventive Health Service *” OR “Primary Prevention” OR empowerment OR “patient participation” OR “Consumer Participation” OR “health promotion” OR “environmental health” OR “public health”) OR SU (“Preventive Health Service *” OR “Primary Prevention” OR empowerment OR “patient participation” OR “Consumer Participation” OR “health promotion” OR “environmental health” OR “public health”)) AND (TI (Nursing OR “community health nursing”) OR AB (Nursing OR “community health nursing”) OR SU (Nursing OR “community health nursing”))	1	1
BASE (Bielefeld Academic Search Engine)	tit: (“climate change” OR “global warming” OR “greenhouse effect”) AND tit: (“Preventive Health Service *” OR “Primary Prevention” OR empowerment OR “patient participation” OR “Consumer Participation” OR “health promotion” OR “environmental health” OR “public health”) AND tit: (Nursing OR “community health nursing)	239	184
RCAAP (through EDS)	(TI (“climate change” OR “global warming” OR “greenhouse effect”) OR AB (“climate change” OR “global warming” OR “greenhouse effect”) OR SU (“climate change” OR “global warming” OR “greenhouse effect”)) AND (TI (“Preventive Health Service *” OR “Primary Prevention” OR empowerment OR “patient participation” OR “Consumer Participation” OR “health promotion” OR “environmental health” OR “public health”) OR AB (“Preventive Health Service *” OR “Primary Prevention” OR empowerment OR “patient participation” OR “Consumer Participation” OR “health promotion” OR “environmental health” OR “public health”) OR SU (“Preventive Health Service *” OR “Primary Prevention” OR empowerment OR “patient participation” OR “Consumer Participation” OR “health	4	4

	promotion" OR "environmental health" OR "public health")) AND (TI (Nursing OR "community health nursing") OR AB (Nursing OR "community health nursing") OR SU (Nursing OR "community health nursing"))

File S1. Ineligible Articles Following Full-Text Review

1. CANTU, A. G. "I Thought It Was Just About Heat": Using the Community as Partner Model to Support Climate Change Education. **Nurse Educator**, *[s. l.]*, v. 49, n. 2, p. 116–117, 2024.

   Reason for exclusion: Full-text not available

2. Gumabay, E.M.S., Ramirez, R.C.,Dimaya, J.M.M., Beltran, M.M. Adversity of prolonged extreme cold exposure among adult clients diagnosed with coronary artery diseases: a primer for recommending community health nursing intervention. Wiley-Blackwell Publishing Ltd. 2017.

   Reason for exclusion: Ineligible context (rural)

3. Koch A. Teach About Heat: Rising Temperature from Climate Change Is a Nursing Issue. J Contin Educ Nurs. 2022 Oct;53(10):460-464. Epub 2022 Oct 1. PMID: 36178761.

   Reason for exclusion: Full-text not available

4. Gerardo Sanchez Martinez, Vladimir Kendrovski, Miguel Antonio Salazar, Francesca de'Donato, Melanie Boeckmann,Heat-health action planning in the WHO European Region: Status and policy implications, Environmental Research, Volume 214, Part 1, 2022, 113709, ISSN 0013-9351.

   Reason for exclusion: Ineligible population
